# Winning the arms race: host–parasite shared evolutionary history reduces infection risks in fish final hosts

**DOI:** 10.1098/rsbl.2018.0363

**Published:** 2018-07-25

**Authors:** Danny J. Sheath, Jaimie T. A. Dick, James W. E. Dickey, Zhiqiang Guo, Demetra Andreou, J. Robert Britton

**Affiliations:** 1 Department of Life and Environmental Sciences, Faculty of Science and Technology, Bournemouth University, Poole, UK; 2 Institute of Global Health, University of Geneva, Geneva, Switzerland; 3 Institute for Global Food Security, School of Biological Sciences, Queen's University Belfast, Medical Biology Centre, 97 Lisburn Road, Belfast BT9 7BL, UK; 4 State Key Laboratory of Marine Resource Utilization in South China Sea, College of Oceanology, Hainan University, Haikou 570228, People's Republic of China

**Keywords:** trophic transmission, parasite manipulation, behaviour, comparative functional response

## Abstract

Parasite manipulation of intermediate hosts evolves to increase parasite trophic transmission to final hosts, yet counter selection should act on the final host to reduce infection risk and costs. However, determining who wins this arms race and to what extent is challenging. Here, for the first time, comparative functional response analysis quantified final host consumption patterns with respect to intermediate host parasite status. Experiments used two evolutionarily experienced fish hosts and two naive hosts, and their amphipod intermediate hosts of the acanthocephalan parasite *Pomphorhynchus tereticollis*. The two experienced fish consumed significantly fewer infected than non-infected prey, with lower attack rates and higher handling times towards the former. Conversely, the two naive fish consumed similar numbers of infected and non-infected prey at most densities, with similar attack rates and handling times towards both. Thus, evolutionarily experienced final hosts can reduce their infection risks and costs via reduced intermediate host consumption, with this not apparent in naive hosts.

## 1. Introduction

Final hosts and their parasites are involved in an evolutionary arms race, whereby trophically transmitted parasites manipulate their intermediate hosts to increase transmission rates, but with final hosts presumably experiencing selection to minimize the risks of infection and thus subsequent fitness costs [1,2]. There is an energetic cost to this selection that needs balancing against other energy demands (e.g. life-history trade-offs) [3–5]. As selection is likely to favour hosts reducing their costs of infection [4], final host populations could evolve behaviours that reduce their ingestion of prey infected with tropically transmitted parasites [4,5]. Moreover, the development of these adaptive behaviours might be influenced by the host's previous experience of the parasite, with experienced hosts likely to elicit stronger anti-parasite responses than naive hosts due to the presence/absence of shared eco-evolutionary histories [6].

Assessing whether final hosts or their parasites are ‘winning’ this arms race has proved difficult, perhaps due to limits on experimental techniques. Correspondingly, we propose that comparative functional responses (CFRs), which compare prey consumption rate as functions of prey density [7–9], can assess the outcome of this arms race. Here, the trophically transmitted acanthocephalan parasite *Pomphorhynchus tereticollis* was the model parasite. It has an amphipod intermediate host and fish final host [10–12]. To test the outcome of selection in final hosts according to their evolutionary history with the parasite, infected and non-infected amphipods were exposed to two parasite-experienced hosts, native chub *Squalius cephalus* and European barbel *Barbus barbus*, and two naive, parasite-inexperienced hosts, non-native goldfish, *Carassius auratus* and carp *Cyprinus carpio*.

## 2. Material and methods

Amphipods used in the experimental feeding trials were from the River Avon, Southern England (latitude: 50.8865, longitude: −1.7883). In this river, *S. cephalus* and *B. barbus* are final hosts of *P. tereticollis* [13], *Ca. auratus* is not present and only small numbers of non-recruiting, large-bodied *Cy. carpio* are present via escapees from adjacent lakes. Infected amphipods were identified visually by the presence of an orange spot [10], with parasitized individuals all at the infectious cystacanth stage, as validated by dissection of 30 visually detected individuals (100% correct).

The experimental fish were sourced from an aquaculture site in Southern England where *P*. *tereticollis* was absent from rearing ponds. Amphipods collected by kick-sampling in May 2018 from the stream upstream of the aquaculture site revealed 0% parasite prevalence (*N* = 200). Concomitantly, samples collected from the River Avon site had a prevalence of 11% (*N* = 200), typical for the time of year, but low compared to later in summer when much higher prevalences are typically recorded [13]. The experimental fish had, therefore, not been exposed to the parasite during husbandry. However, the broodstock of *S. cephalus* and *B. barbus* were from the River Kennet, a tributary of the River Thames, southeast England, where the fishes and parasite are native and coexist. Correspondingly, *S. cephalus* and *B. barbus* were used as the parasite-experienced final hosts. Conversely, the broodstock of *Ca. auratus* and *Cy. carpio* had no known previous experience of the parasite and thus were used as the naive hosts. Both species can, nevertheless, develop *Pomphorhynchus* parasite infections [14,15]. All fish were 60–80 mm length and prior to use were individually tagged (7 mm passive integrated transponder tag), and acclimated for 20 days (18°C; 16 L : 8 D cycle).

For CFR experiments, individual fish were exposed to either infected or non-infected amphipods as prey in 10 l tanks at 18°C following a 24 h starvation period. Prey densities were 4, 8, 16, 32 and 64 amphipods [16]. Prey exposure was for 1 h, with three replicates per prey density [8]. Values of the CFR parameters attack rate (*a*) and handling time (*h*) were calculated using maximum-likelihood estimation in the random predator equation [17], completed in the R package *Frair* [16]. The equation assumes a type II functional response and the non-replacement of prey, where *N*_e_ = *N*_0_ (1 – exp(*a*(*N*_e_*h*−*T*))), where *N*_e_ is the number of prey eaten, *N*_0_ the initial density of prey, *a* the attack rate, *h* the handling time and *T* the total time. Analyses also provided the significance of differences in *a* and *h* between the prey types [16]. To visualize uncertainty, 2000 non-parametric bootstraps enabled empirical 95% confidence intervals to be fitted around the functional responses. These were used to provide CFR plots between the infected/non-infected amphipods per fish host [16]. Following experiments, the fish were euthanized and their infection status and parasite loading determined.

## 3. Results

**Figure 1.**
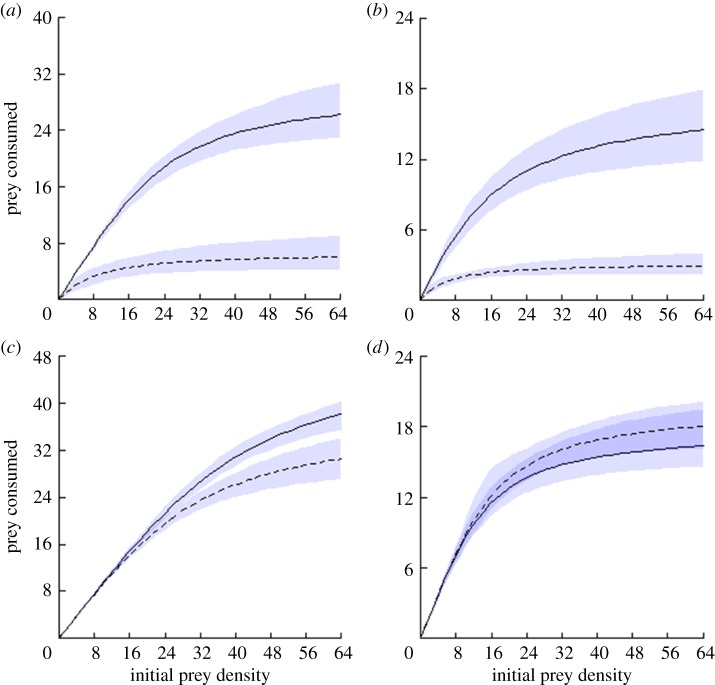
Type II functional response curves for experienced final hosts ((*a*) *S. cephalus*, (*b*) *B. barbus*) and naive hosts ((*c*) *Ca. auratus*, (*d*) *Cy. carpio*) fed infected (dashed line) and non-infected (solid line) amphipods. Lines indicate the type II functional response, shading represents 95% equi-tailed confidence intervals (CI) for each combination of fish and prey [15]. Consumption rates in numbers of amphipods consumed per hour. Note differences in *y*-axis values. (Online version in colour.)

The CFR curves of the two experienced fish final hosts revealed significantly lower consumption rates of infected versus non-infected amphipods (figure 1*a,b*). This was driven by lower attack rates and higher handling times towards infected amphipod prey (table 1).

**Table 1.** Functional response parameters of infected versus non-infected amphipods per final host and the significance of their differences.

	experienced hosts		naive hosts	
	*Squalius cephalus*	*Barbus barbus*	*Carassius auratus*	*Cyprinus carpio*
*a*	1.06/4.08	0.64/1.73	3.52/3.79	3.65/3.62
*p-*value	<0.01	0.07	0.71	0.98
*h*	1.15/0.03	0.32/0.06	0.03/0.04	0.05/0.06
*p-*value	<0.01	<0.01	0.04	0.45

On the other hand, for the two naive, fish final hosts, attack rates and handling times between infected and non-infected amphipods were similar (table 1). The *Ca. auratus* CFR curves indicated differences between prey types were minimal at low prey densities (e.g. mean consumption rate at 32 items (±95% confidence limits): 24.7 ± 3.0/23.0 ± 4.2 n.h^−1^) and only at the highest prey densities did consumption rates of infected amphipods decrease versus non-infected (figure 1*c*). In *Cy. carpio*, their CFR curves overlapped completely for infected and non-infected prey (figure 1*d*).

All fish that consumed infected amphipods developed infections. In experienced hosts, parasite loadings were one to five adults in *S. cephalus* and one adult in *B. barbus*. In naive hosts, parasite loadings were eight to 27 adults in *Ca. auratus* and eight to 15 adults in *Cy. carpio*. Higher parasite loadings occurred in fish that consumed more infected amphipods. In some *Ca. auratus*, parasites had perforated the intestine and were embedded in muscle tissue.

## 4. Discussion

The evolutionary arms race between trophically transmitted parasites and their final hosts involves the interaction of the parasites manipulating the behaviours of their intermediate hosts versus potential final hosts minimizing their infection risk and fitness costs [1–5]. The CFRs revealed the outcome of this arms race was strongly dependent on whether there was a shared evolutionary history in the parasite–final host system, with contrasting outcomes for experienced and naive hosts.

For naive hosts, the CFR curves revealed similar consumption rates of infected and non-infected prey. This was consistent with other studies suggesting infected amphipods are preferred to uninfected by fish hosts due to parasite manipulation [1]. The consumption by naive hosts of relatively high numbers of infected amphipods resulted in consistently high parasite loadings. The associated host pathology, including intestinal perforation, suggested high energetic and fitness costs. This apparent low avoidance of infection [5,6] was interpreted as largely due to their lack of previous experience of the parasite. Conversely, for experienced fish hosts, CFR curves revealed significantly reduced consumption rates of infected versus non-infected amphipods. Although fish that consumed infected prey developed infections, parasite loadings were relatively low. These results strongly suggest the experienced hosts used a range of anti-parasite responses that reduced their risks of developing high parasite loadings by minimizing their exposure to infected prey [3–5]. The results suggested the mechanism of experienced fishes avoiding consumption of infected prey was their lower ‘attack’ or ‘encounter’ rates towards the parasite-manipulated prey. Further, the high handling times of infected prey suggest some mechanism of prey assessment and selection, and this warrants detailed quantification of the behaviour and sensory modes involved, and in relation to parasite manipulation [1,2].

Although the significant differences in CFRs between the two fish host groups were interpreted as being due to their differing parasite experience, a potential confound was trait differences relating to habitat preferences of experienced (lotic) and naive (lentic) hosts. However, amphipods are naturally present in both habitats, and are active in the benthos and water column, thus naturally feature in the diets of all hosts [18]. Thus, any trait-mediated differences in fish foraging behaviours were considered as not influencing the ability of the fishes to consume amphipod prey and were not confounding the experiment.

Behavioural responses to the infective stages of parasites by potential hosts have generally resulted in reduced infection levels in host populations. Increased activity in *Rana* spp. tadpoles occurred in the presence of a number of parasite species that successfully reduced infection risk [19]. In exposure experiments on Pacific chorus frogs *Pseudacris regilla*, hosts with inhibited anti-parasite behaviours had higher parasite prevalences, with inhibited individuals having higher parasite loadings [20]. These studies suggested host behaviours were more effective at reducing infection risk than immune-mediated responses. Indeed, avoidance of *Diplostomum spathaceum* by rainbow trout *Oncorhynchus mykiss* was stronger through avoidance of infection sources than from physiological resistance gained from previous exposure [21]. These studies suggest the behaviour of our experienced hosts was a selection mechanism to reduce infection risk and costs by minimizing their consumption of infected amphipods. Although this successfully reduced their parasite loadings compared with naive hosts, the parasite was still transmitted to experienced hosts, enabling life cycle completion. Thus, even in experienced hosts, infection risk and costs are only reduced, not eliminated. The extent of the balancing of the evolutionary arms race between host and parasite has thus been revealed by our approach and this should prove fruitful in future studies.

## Supplementary Material

Sheath_et_al_data

## Data accessibility

Data are available in the electronic supplementary material file.
